# Effect of Cardiotonic Steroid Marinobufagenin on Vascular Remodeling and Cognitive Impairment in Young Dahl-S Rats

**DOI:** 10.3390/ijms23094563

**Published:** 2022-04-20

**Authors:** Yulia N. Grigorova, Ondrej Juhasz, Jeffrey M. Long, Valentina I. Zernetkina, Mikayla L. Hall, Wen Wei, Christopher H. Morrell, Natalia Petrashevskaya, Audrey Morrow, Katherine H. LaNasa, Alexei Y. Bagrov, Peter R. Rapp, Edward G. Lakatta, Olga V. Fedorova

**Affiliations:** 1 Laboratory of Cardiovascular Science, National Institute on Aging, NIH, Baltimore, MD 21224, USA; yulia.grigorova2@nih.gov (Y.N.G.); juhaszo@grc.nia.nih.gov (O.J.); valentina.zernetkina@nih.gov (V.I.Z.); hallx810@umn.edu (M.L.H.); wen.wei@nih.gov (W.W.); christopher.morrell@nih.gov (C.H.M.); voitsat@gmail.com (N.P.); aybagrov@gmail.com (A.Y.B.); lakattae@grc.nia.nih.gov (E.G.L.); 2 Laboratory of Behavioral Neuroscience, Neurocognitive Aging Section, National Institute on Aging, NIH, Baltimore, MD 21224, USA; longjm@mail.nih.gov (J.M.L.); morrow.a.90@gmail.com (A.M.); schulze.kh@gmail.com (K.H.L.); rappp@mail.nih.gov (P.R.R.)

**Keywords:** arterial stiffness, behavioral tests, cardiovascular remodeling, Dahl salt-sensitive rats, echocardiography, fibrosis, genes, hypertension, marinobufagenin, aortic pulse wave velocity, spatial memory, steroidal inhibitor of Na/K-ATPase, vascular dementia

## Abstract

The hypertensive response in Dahl salt-sensitive (DSS) rats on a high-salt (HS) diet is accompanied by central arterial stiffening (CAS), a risk factor for dementia, and heightened levels of a prohypertensive and profibrotic factor, the endogenous Na/K-ATPase inhibitor marinobufagenin (MBG). We studied the effect of the in vivo administration of MBG or HS diet on blood pressure (BP), CAS, and behavioral function in young DSS rats and normotensive Sprague–Dawley rats (SD), the genetic background for DSS rats. Eight-week-old male SD and DSS rats were given an HS diet (8% NaCl, *n* = 18/group) or a low-salt diet (LS; 0.1% NaCl, *n* = 14–18/group) for 8 weeks or MBG (50 µg/kg/day, *n* = 15–18/group) administered via osmotic minipumps for 4 weeks in the presence of the LS diet. The MBG-treated groups received the LS diet. The systolic BP (SBP); the aortic pulse wave velocity (aPWV), a marker of CAS; MBG levels; spatial memory, measured by a water maze task; and tissue collection for the histochemical analysis were assessed at the end of the experiment. DSS-LS rats had higher SBP, higher aPWV, and poorer spatial memory than SD-LS rats. The administration of stressors HS and MBG increased aPWV, SBP, and aortic wall collagen abundance in both strains vs. their LS controls. In SD rats, HS or MBG administration did not affect heart parameters, as assessed by ECHO vs. the SD-LS control. In DSS rats, impaired whole-heart structure and function were observed after HS diet administration in DSS-HS vs. DSS-LS rats. MBG treatment did not affect the ECHO parameters in DSS-MBG vs. DSS-LS rats. The HS diet led to an increase in endogenous plasma and urine MBG levels in both SD and DSS groups. Thus, the prohypertensive and profibrotic effect of HS diet might be partially attributed to an increase in MBG. The prohypertensive and profibrotic functions of MBG were pronounced in both DSS and SD rats, although quantitative PCR revealed that different profiles of profibrotic genes in DSS and SD rats was activated after MBG or HS administration. Spatial memory was not affected by HS diet or MBG treatment in either SD or DSS rats. Impaired cognitive function was associated with higher BP, CAS, and cardiovascular remodeling in young DSS-LS rats, as compared to young SD-LS rats. MBG and HS had similar effects on the cardiovascular system and its function in DSS and SD rats, although the rate of change in SD rats was lower than in DSS rats. The absence of a cumulative effect of increased aPWV and BP on spatial memory can be explained by the cerebrovascular and brain plasticity in young rats, which help the animals to tolerate CAS elevated by HS and MBG and to counterbalance the profibrotic effect of heightened MBG.

## 1. Introduction

The Dahl salt-sensitive (DSS) rat model of salt-sensitive hypertension is characterized by cardiovascular remodeling and the development of central arterial stiffness (CAS) associated with a high-salt (HS) diet [1,2,3,4]. DSS rats were selected from Sprague–Dawley (SD) rats by backcrossing for the trait of salt sensitivity to blood pressure (BP) [5,6]. DSS rats have a genetically determined impaired ability to excrete sodium with HS intake [7,8], a scenario that requires a higher level of natriuretic factors. It was demonstrated that HS intake in DSS rats stimulates the production of bufadienolide, a steroidal natriuretic hormone marinobufagenin (MBG), in response to sodium retention [4,9]. MBG inhibits the sodium pump in renal tubules to promote natriuresis in volume-expanded states, i.e., salt-sensitive hypertension [9,10], preeclampsia [11], and renal failure [12]. However, excessive MBG production causes inhibition of vascular Na/K-ATPase (NKA) and potentiates vasoconstriction [13], leading to a BP increase.

MBG is associated with preferential inhibition of the sodium pump in vascular smooth muscle due to higher sensitivity of NKA to MBG, resulting in a greater hypertensive response in DSS rats in comparison to SD rats [2]. MBG is also implemented in the development of cardiac and renal fibrosis and arterial stiffening in DSS rats via TGFβ-dependent and Fli-1-dependent profibrotic pathways [3,14,15]. The profibrotic effect of MBG was demonstrated by exogenous infusion of this steroid to SD rats, which caused a moderate systolic blood pressure (SBP) increase, the development of left ventricle (LV) hypertrophy, and cardiac fibrosis [14,15]. In addition, a TGFβ-dependent MBG-mediated increase in aortic fibrosis and CAS was demonstrated in normotensive SD rats with HS intake [10,16,17].

Salt-induced hypertension in DSS rats leads to cardiac remodeling, resulting in congestive heart failure [3,16], cardiovascular and renal fibrosis [3,17], and stroke [18,19,20]. Cardiovascular diseases (CVD) and hypertension are major contributors to the pathogenesis of vascular dementia [21,22,23,24]. Because the profound aortic stiffening and hemodynamic alterations are associated with cognitive impairment [21,22,23,24,25,26,27,28], CAS is a potential contributor to the development of age-associated microvascular disease and cognitive impairment. Few studies have been conducted to investigate cognitive function in DSS rats. DSS rats exhibit cognitive dysfunction at a young age, even with a low salt (LS) intake [29,30]. The Morris water maze (MWM) behavioral test detected significant differences in spatial learning and memory between Dahl salt-sensitive and Dahl salt-resistant (DSR) rats [29,31]. The passive avoidance test revealed cognitive decline in young DSS rats with HS intake vs. LS intake, which was ameliorated by antihypertensive treatment [30]. In contrast, salt restriction in DSS rats vs. their DSR counterparts did not affect spatial memory but impaired social recognition memory [32]. Although the association between BP, CAS, and cognitive impairment has been recognized in epidemiological and animal model studies, the mechanistic links between these parameters are not fully understood. Moreover, such mechanistic links between cognitive function and vascular structure/function have not yet been explored in animal models. The DSS rat model may represent a model of vascular dementia [29,30,31,32], but studies using the DSS model are scarce.

In the present study, we compared cardiovascular remodeling and behavioral performance in young DSS and SD rats with LS and HS intake and investigated whether the administration of MBG, a profibrotic and prohypertensive factor, has a similar effect to HS administration, and whether MBG affects BP and aPWV and activates the profibrotic pathways involved in cardiovascular remodeling. We hypothesized that higher CAS will be associated with cognitive impairment in DSS rats vs. SD rats, and that the administration of the stressors MBG and HS would result in different profiles of cardiovascular changes and profibrotic gene expression in normotensive and hypertensive rats.

## 2. Results

**Figure 1:**
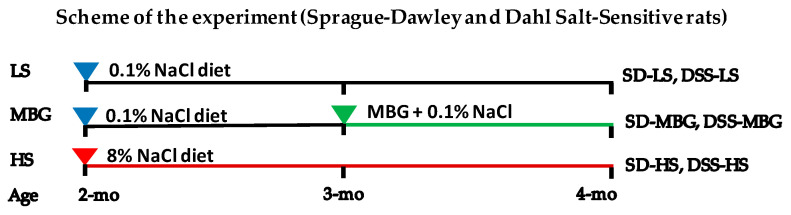
Schematic presentation of the experimental design and the group description. Sprague–Dawley (SD) and Dahl salt-sensitive (DSS) rats received a low-salt (LS) diet for 8 weeks, a high-salt (HS) diet for 8 weeks, or marinobufagenin (MBG), which was administered via osmotic minipumps for 4 weeks. The MBG-treated rats received an LS diet for the duration of the study.

The following six groups of rats were studied: SD-LS, SD-MBG, SD-HS, DSS-LS, DSS-MBG, and DSS-HS. A detailed description of the experimental design is presented in the Methods section and Figure 1.

First, we compared physiological, biochemical, and behavioral parameters, obtained from young 4-month-old SD rats and DSS rats subjected to LS intake. Second, we compared the effect of the stressors, i.e., MBG or an HS diet, on the above parameters in both strains.

### 2.1. Young DSS Rats on an LS Diet Have Higher aPWV/CAS and Impaired Spatial Memory in Comparison to SD Rats on an LS Diet

#### 2.1.1. Difference between DSS Rats and SD Rats in Physiological and Biochemical Parameters with Low Salt (LS) Intake

Most of the measured parameters in SD-LS and DSS-LS rats are presented in the Table 1 and Table 2, where they were used as the non-treated control groups. Four-month-old DSS-LS rats exhibited lower body weight, higher SBP, and higher heart rate (HR) in comparison to the age-matched SD-LS rats. DSS-LS rats also had a higher plasma creatinine concentration vs. their SD-LS counterparts. Notably, plasma MBG and urinary MBG excretion were similar in both strains on an LS intake, whereas brain MBG concentration was significantly lower in DSS-LS rats in comparison to SD-LS rats (Table 1).

The cardiovascular parameters, obtained by ECHO, are presented in Table 2. aPWV, fractional shortening (FS), and ejection fraction (EF) were higher in DSS-LS vs. SD-LS rats. LV internal diameter in systole and diastole (LVIDd and LVIDs) were smaller in DSS-LS vs. SD-LS rats. The LV and aortic weights, adjusted for body weight, were higher in DSS-LS in comparison to SD-LS rats (Table 2). These findings indicate the presence of cardiovascular disparity between young four-month-old DSS rats and SD rats when both groups were kept on an LS diet.

#### 2.1.2. Difference between SD Rats and DSS Rats in Expression of Fibrosis and Alzheimer’s Disease Genes in the Left Ventricle (LV)

DSS rats from the DSS-LS group exhibited higher levels of LV mRNA related to the regulation of profibrotic gene expression, i.e., *Col1α2*, *Col3α1*, *Col4α1*, *Col5α2*, *Ctgf* and *FN-1*, vs. those in SD-LS (Table 3). *Fli-1* mRNA was lower in DSS-LS vs. SD-LS by 20%. Fli-1 is a nuclear factor and a negative regulator of the biosynthesis of collagen. Thus, Fli-1 is a profibrotic factor [33,34]. The higher level of *APOE* mRNA in LV in DSS-LS rats may be an indicator of an increase in the cell repair process, which may accompany cardiovascular and metabolic abnormalities [35]. DSS-LS rats demonstrated more than two-fold higher levels of amyloid precursor protein *APP* mRNA in LV vs. SD-LS rats (Table 3). *PSEN-1* and *PSEN-2* mRNAs were expressed at the same levels in both strains on an LS diet.

#### 2.1.3. Difference between SD Rats and DSS Rats in Morris Water Maze Behavioral Test

**Figure 2:**
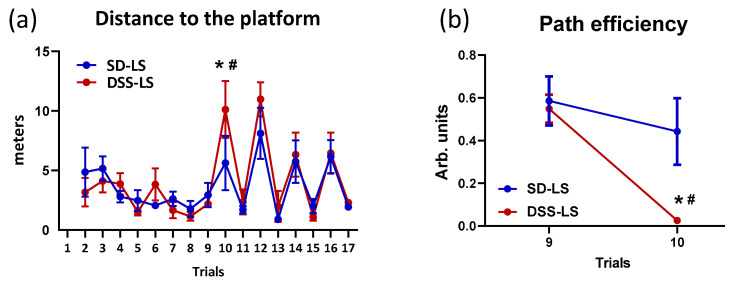
Morris water maze (MWM) behavioral test results. MWM test was performed in four-month-old Sprague–Dawley (SD; *n* = 8; blue line) rats and four-month-old Dahl salt-sensitive (DSS; *n* = 7; red line) rats, which were kept on a low-salt (LS) diet. Values are expressed as mean ± SEM. (**a**) Distance to the platform in affected MWM test. Visible platform: trials 1–9, 11, 13, 15, and 17; invisible platform: trials 10, 12, 14, and 16. (**b**) Path efficiency in MWM. The differences between trials 9 (visible platform) and 10 (invisible platform) are presented. Two-way ANOVA with false discovery rate (FDR) correction: * *p* < 0.01, for trial 9 vs. trial 10; # *p* < 0.05, for SD vs. DSS. The DSS rats traveled longer distances to find the invisible platform vs. visible platform in comparison to the SD rats.

Animals were tested on a redundant place-cue version of the water maze in which they initially swim to a visible platform in a fixed location across trials. Rats can solve these trials using either a non-spatial goal approach strategy, or by learning the location of the platform relative to extra-maze cues, i.e., a capacity that requires the integrity of the hippocampus (for details, see Section 5.5). Both SD-LS and DSS-LS rats traveled the same distance to reach a visible platform during training (trials 2–9) in the MWM test (Figure 2a). To assess the extent that rats encoded the location of the platform via spatial cues, the platform was hidden under the water on trials 10, 12, 14 and 16 (Figure 2a). On the first nonvisible platform trial (trial 10), DSS-LS rats traveled longer distances to the platform than in trial 9, specifically, the last trial with the visible platform in comparison to SD-LS, which did not exhibit difference in distance between trials 9 and 10 (Figure 2a). The transfer between trials 9 and 10 means learning ability and memory for the spatial location of the escape platform. The DSS-LS rats exhibited poorer performance in this task vs. SD-LS rats. To note, no difference in travelled distance was observed in trials 11–17 between DSS and SD rats. Path efficiency is a ratio of the optimal path to the platform to the actual route taken by the individual rat (Figure 2b). This ratio was used to quantify the efficiency of the strategy pursued in reaching the platform. It was similar in the SD-LS and DSS-LS groups in trial 9, with the visible platform, and became significantly worse in DSS-LS rats in trial 10, when the platform was not visible for the first time (Figure 2b). The SD-LS did not exhibit differences in path efficiency between trials 9 (visible platform) and 10 (invisible platform) (Figure 2b).

#### 2.1.4. Difference between SD Rats and DSS Rats in Density of Neurons in Hippocampus

**Figure 3:**
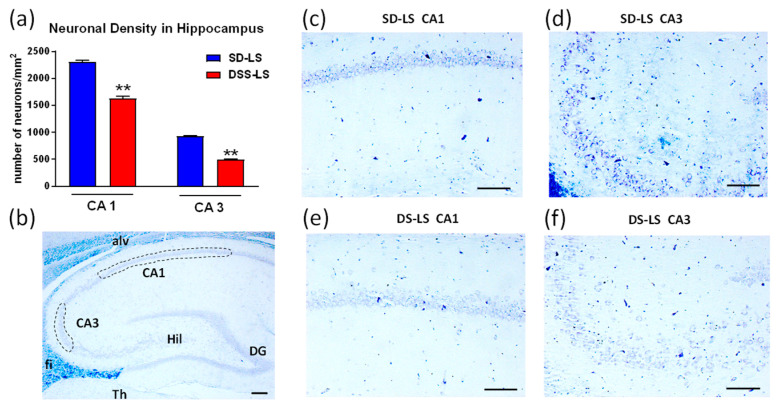
Neuronal density in the hippocampus of Sprague–Dawley (SD-LS) and Dahl salt-sensitive (DSS-LS) rats which were kept on a low-salt (LS) diet. (**a**) Neuronal density in CA1 and CA3 regions of hippocampus is presented as estimated number of neurons per mm^2^ in a particular hippocampus region. Bars are presented as mean ± SEM; unpaired Student’s *t*-test: ** *p* < 0.01, DSS-LS rats (*n* = 5) vs. SD-LS rats (*n* = 5). (**b**) Representative image of a sagittal section of rat brain stained by Luxol/Cresyl violet stain; scale bar is 200 μm (right bottom corner). The quantified CA1 and CA3 regions which were used for the analysis of the neuronal density are schematically outlined by the dotted lines. Abbreviations for hippocampus and the surrounding regions: alv, alveus layer of CA; CA1 and CA3, Cornu Ammonis fields 1 and 3; DG, dentate gyrus; fi, fimbria; Hil, Hilus of DG; Th, thalamus. (**c**–**f**) Representative photomicrographs of CA fields stained by Luxol/Cresyl violet stain. Neurons are stained dark blue to purple. Scale bars, 50 μm (right bottom corner). (**c**) SD-LS CA1 area; (**d**) SD-LS CA3 area; (**e**) DSS-LS CA1 area, (**f**) DSS-LS CA3 area.

The hippocampus is a brain region critical for normal memory function, including spatial memory [36]. The density of neurons was lower in the CA3 region vs. the CA1 region in both rat strains, which is in agreement with normal hippocampal anatomy (Figure 3a). Rats from the DSS-LS group demonstrated a significantly lower density of neurons in both the CA1 and CA3 regions of the hippocampus in comparison to the SD-LS group (Figure 3a,c–f). The neuronal count represented the sum of pyramidal neurons and neuronal cell bodies in the selected fields, presented in Figure 3b, and was expressed as the number of neurons per mm^2^ of the measured area. We further found that hippocampal neuronal density was affected neither by MBG nor by HS, in both SD and DSS rats in comparison to their LS controls (data are not presented).

The abundance of collagen in the medial part of the arterial wall in the cerebral arteries did not differ between the SD-LS and DSS-LS rats (Supplementary Materials, Figure S2). The collagen accumulation in the cerebral arteries was not affected by MBG in both SD and DSS rats, and by HS in SD in comparison to the LS control groups. Significant collagen abundance was observed only in DSS-HS group vs. DSS-LS (Supplementary Materials, Figure S2).

### 2.2. Effect of the Stressor Agents, MBG or High-Salt Diet, in Young SD and DSS Rats

#### 2.2.1. Effect of MBG or an HS Diet on Physiological and Biochemical Parameters in SD Rats and DSS Rats

**Figure 4:**
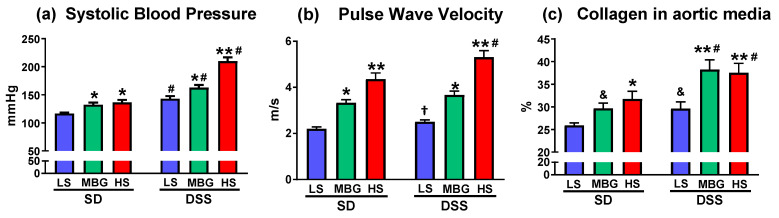
Effect of administration of MBG and high-salt (HS) dietary intervention on systolic blood pressure (**a**), pulse wave velocity (**b**), and collagen abundance in aortic wall (**c**). Bars represent mean ± SEM. Two-way ANOVA followed by Holm-Sidak’s multiple comparison post hoc test: * *p* < 0.05, ** *p* < 0.01 vs. LS groups for both SD and DSS rats; # *p* < 0.01 DSS vs. SD; & 0.05 < *p* < 0.1, vs. SD-LS. Two-tail unpaired *t*-test: † *p* < 0.05, DSS-LS vs. SD-LS (pulse wave velocity). Experimental group: LS, low salt for 8 weeks (blue bars); MBG, administration of MBG for 4 weeks (green bars); HS, high salt for 8 weeks (red bars); SD, Sprague–Dawley rats; DSS, Dahl salt-sensitive rats.

**Figure 5:**
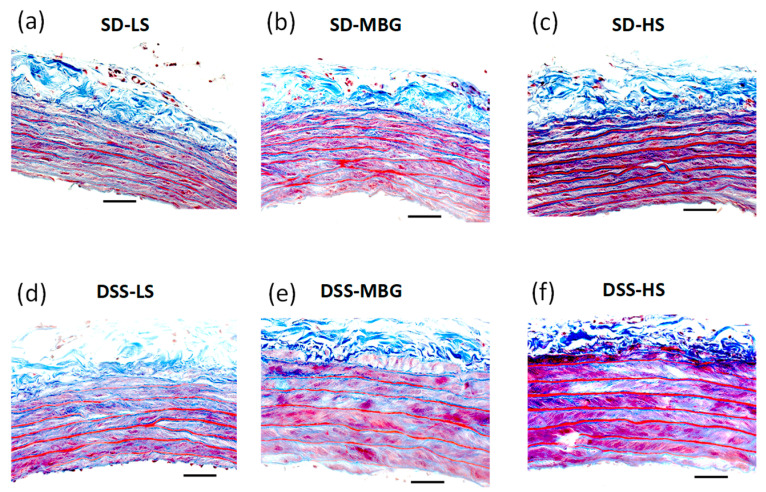
(**a**–**f**) Representative photomicrographs of aortae from each experimental group stained with Masson’s trichrome stain. Collagen in the aortic wall is stained blue; muscle and intracellular fibers are magenta. SD-LS (**a**), SD-MBG (**b**), SD-HS (**c**), DSS-LS (**d**), DSS-MBG (**e**), DSS-HS (**f**). SD, Sprague–Dawley rats; DSS, Dahl salt-sensitive rats; LS, low-salt diet; MBG, marinobufagenin; HS, high-salt diet. Scale bar is 50 μm.

DSS-LS rats had significantly lower body weight (BW) in comparison to SD-LS rats. MBG treatment did not affect BW in either strain. HS diet significantly affected BW in both strains (Table 1). SBP was higher in DSS-LS vs. SD-LS rats (Figure 4a, Table 1). Administration of MBG was associated with a moderate SBP elevation in SD-MBG vs. SD-LS and a pronounced hypertensive effect in DSS-MBG vs. DSS-LS rats. aPWV was higher after MBG administration in both strains vs. corresponding groups on an LS intake (green bars vs. blue bars, Figure 4a,b). An HS diet resulted in an SBP elevation and an aPWV increase in both strains vs. LS (red bars vs. blue bars, Figure 4a,b). Notably, the hypertensive effect of HS intake was more pronounced in DSS-HS than in SD-HS rats (Figure 4a; Table 1), reaching values for SBP over 200 mmHg, which is expected in the DSS rats upon HS intake, as was demonstrated in previous studies [1,3]. Both SD and DSS rats, which were administered MBG or an HS diet, exhibited higher collagen abundance in the aortic wall vs. the LS controls (Figure 4c), which is in congruence with aPWV changes (Figure 4b). The representative photomicrographs of aortae from each experimental group are presented in Figure 5. Notably, that the difference in aortic collagen in SD-MBG vs. SD-LS, and in DSS-LS vs. SD-LS was borderline significant by two-way ANOVA analysis (Figure 4c).

Plasma MBG was similar in both SD-LS and DSS-LS groups on an LS diet and increased after MBG or HS intake in both SD and DSS groups vs. LS controls (Table 1). Plasma MBG doubled in DSS-MBG rats after MBG administration and increased 2.8-fold after HS diet intervention in DSS-HS vs. DSS-LS rats. In the SD rats, plasma MBG increase was moderate in SD-MBG and SD-HS vs. SD-LS (by *t*-test, Table 1). Urine MBG excretion was similar in both SD-LS and DSS-LS groups. MBG administration moderately increased urine MBG in both SD-MBG and DSS-MBG vs. LS groups (by *t*-test). HS intake was associated with a more dramatic increase in urine MBG in both strains, i.e., with a three-fold increase in urine MBG in SD-HS rats vs. SD-LS rats, and with a six-fold increase in MBG in urine in DSS-HS vs. DSS-LS rats (Table 1). Interestingly, in the present study, brain MBG was 2.5-fold lower in DSS-LS rats vs. SD-LS rats, and significantly increased after MBG or HS administration in both strains vs. their LS controls (Table 1).

Plasma sodium, plasma potassium, and hematocrit did not differ between strains and treatments, except for hematocrit in the DSS-HS group, which was significantly lower in comparison to the other groups (Table 1). This decrease in hematocrit is consistent with previous observations obtained in DSS rats with HS intake [37], and it is an indication of the relative volume expansion due to compromised renal function, which is developed under conditions of HS intake [38]. Plasma creatinine was higher in all three DSS groups vs. corresponding SD groups. MBG and HS did not affect plasma creatinine in SD rats. In DSS rats, only HS intervention increased plasma creatinine vs. DSS-LS rats. Blood urea nitrogen (BUN) did not differ between SD-LS and DSS-LS groups, but it was higher in DSS-MBG vs. SD-MBG and in DSS-HS vs. DSS-HS groups. BUN was also increased in DSS-HS vs. DSS-LS groups. The volume of urine did not differ between strains with LS intake or after MBG administration. HS intake affected the volume of urine in both strains vs. that in the LS groups, and it was significantly higher in DSS-HS vs. SD-HS rats. Creatinine clearance was significantly lower in the DSS-HS group, only in comparison to DSS-LS and to SD-HS rats, which is an indication of compromised renal function due to an HS diet (Table 1).

ECHO parameter data are presented in Table 2. In SD, MBG or HS intake affected aPWV only: this parameter was higher in SD-MBG and SD-HS vs. SD-LS. In DSS rats, MBG was associated with an aPWV increase. HS intake resulted in changes to multiple parameters in DSS-HS vs. DSS-LS rats, i.e., rats in this group had a thicker interventricular septum at end diastole and systole, thicker LV posterior wall at end diastole and systole, increased LV mass and higher aPWV, smaller LV internal diameter at end diastole, and lower fractional shortening vs. DSS-LS rats (Table 2). These findings are in agreement with previous publications describing the HS effect in DSS rats. High-salt diet induced the development of left ventricle concentric hypertrophy in the DSS-HS group. Reduced myocardial contraction in the DSS-HS group indicated the beginning of the transition to heart failure [1,3]. MBG administration was associated with an increase in aortic weight in both SD-MBG and DSS-MBG groups vs. corresponding LS groups (determine in SD by *t*-test). HS intake was associated with increased aortic weight and kidney weight in both SD-HS and DSS-HS groups vs. the LS group ((in SD: by *t*-test; Table 2).

Both SD and DSS rats administered MBG or an HS diet, exhibited higher collagen abundance in the aortic wall vs. LS controls (Figure 4c), similarly to the aortic weight changes (Table 2, Tissue Weights). Notably, the increase in aortic wall collagen in the SD rats treated with both stressors, i.e., MBG and HS, was moderate in comparison to DSS rats. The representative photomicrographs of the aortae from each experimental group, stained with Masson’s trichrome stain, are presented in Figure 5a–f. Some of the aortic samples were stained with Verhoeff’s stain to detect elastin abundance (Supplementary Figure S1). No difference in the amount of elastin between the SD and DSS strains, nor between the three treatments, i.e., LS, MBG and HS (data not presented), were detected.

#### 2.2.2. Effect of Marinobufagenin (MBG) or a High-Salt (HS) Diet on the Expression of Fibrosis and Alzheimer’s Disease Genes in the Left Ventricle of SD Rats and DSS Rats

The expression of profibrotic genes in LV from the rat groups, estimated by qPCR, is presented in Table 4 and Table 5. The results for the SD-LS, SD-MBG, and SD-HS groups are presented in Table 4, and the results for DSS-LS, DSS-MBG, and DSS-HS groups are presented in Table 5. The effect of MBG and HS on the expression of the genes of interest was calculated as fold change relative to the LS control (Methods, Section 5.9). In SD rats, MBG administration was associated with the upregulation of *TGFβ-1* and *Col1α2* mRNAs and downregulation of *Fli-1* mRNA (Table 4). HS did not affect *Fli-1* in SD-HS, and it upregulated *TGFβ-1*, *Col5α1*, and *Ctgf* mRNAs in SD-HS vs. SD-LS rats (Table 4).

In the DSS rats, both stressors had a stronger impact on the LV mRNA profile (Table 5). MBG administration resulted in the upregulation of *Col1α2*, *Col3α1*, and *Col4α1* mRNAs and dramatic downregulation of *APOE* and *Fli-1* mRNA. HS intake was associated with the upregulation of *TGFβ-1*, *Col1α1*, *Col1α2*, *Col3α1*, *Col4α1*, *Col5α1*, *Col4α2*, *Ctgf*, and *FN-1* mRNAs. *APOE* mRNA was downregulated in DSS-HS vs. DSS-LS rats. Interestingly, MBG also downregulated the genes involved in the Alzheimer’s disease (AD) genomic profile in DSS rats, i.e., *APP* mRNA expression was almost two times lower, and *PSEN-1* mRNA was 40% lower in DSS-MBG vs. DSS-LS rats (Table 5). This phenomenon was not observed in SD, treated with MBG, nor in both strains after HS intervention. Moreover, HS intake was associated with an increase in the expression of *APP* mRNA in DSS-HS vs. DSS-LS rats, which indicated the involvement of the factors stimulated by HS intake.

**Figure 6:**
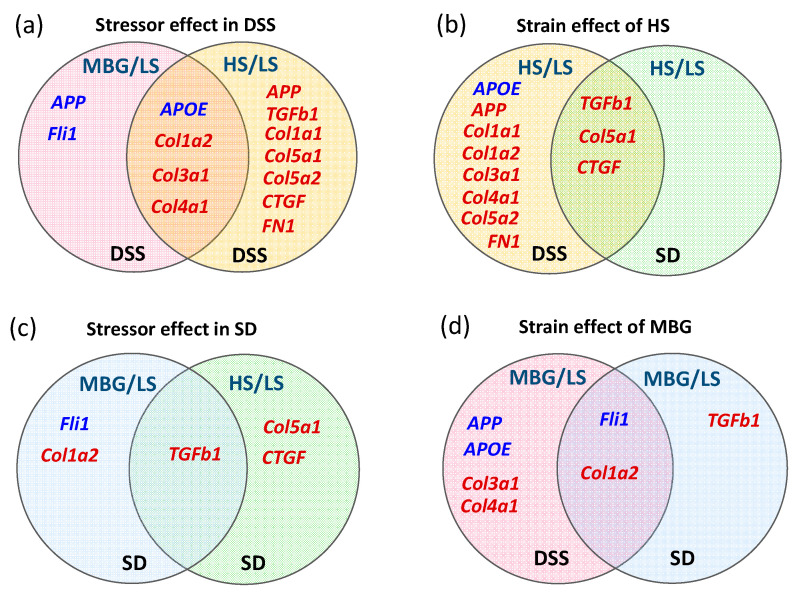
Venn diagrams of the expression of profibrotic and AD-related genes. (**a**) The effect of marinobufagenin (MBG) and high-salt diet (HS) in comparison to low-salt diet (LS) in Dahl salt-sensitive (DSS) rats. (**b**) The effect of the HS in comparison to LS in DSS vs. Sprague–Dawley (SD) rats. (**c**) The effect of MBG and HS in comparison to LS in SD rats. (**d**) The effect of MBG in comparison to LS in DSS vs. SD rats. The graphic presentation is based on the qPCR data (Table 4 and Table 5). The genes that are downregulated in comparison to the LS control are presented in blue and the genes that are upregulated in comparison to LS control are shown in red. The genes in the intersecting areas are shared between the compared groups.

The data on the profibrotic and AD-related gene expression in both strains treated with MBG and HS stressors are summarized in the Venn diagrams in Figure 6. It is likely that in DSS rats, MBG initiated profibrotic signaling via the Fli-1-dependent pathway, whereas HS intake involved TGFβ-1-dependent profibrotic signaling, exhibiting a dissociation between the two stressors in the DSS model. In SD rats, MBG administration was associated with the activation of both Fli-1- and TGFβ-1-dependent profibrotic signaling, whereas the HS intervention was accompanied by the activation of the TGFβ-1 pathway only, similar to DSS rats (Figure 6).

#### 2.2.3. Effect of Marinobufagenin (MBG) or a High-Salt (HS) Diet on Behavioral Test Results in SD Rats and DSS Rats

**Figure 7:**
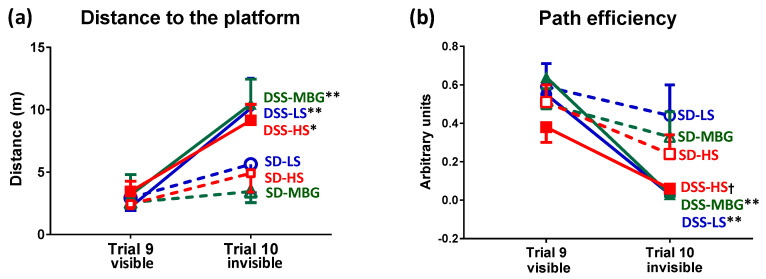
Morris water maze (MWM) test results. (**a**) Distance to the platform in MWM test. (**b**) Path efficiency in MWM test. MWM test was performed in four-month-old Sprague-Dawley (SD) rats and four-month-old Dahl salt-sensitive (DSS) rats which received low-salt (LS) diet (SD, *n* = 8; DSS, *n* = 7), MBG (SD, *n* = 8; DSS, *n* = 7) and high-salt (HS) diet (SD, *n* = 7; DSS, *n* = 8). Values are expressed as mean ± SEM. Two-way ANOVA followed by Tukey’s post hoc test: * *p* < 0.05, ** *p* < 0.01, for trial 9 vs. trial 10 in the corresponding group. Two-tailed *t*-test: † *p* < 0.05 for trial 9 vs. trial 10 in DSS-HS. Dotted lines, SD groups; solid lines, DSS groups; blue, LS; green, MBG; red, HS.

As presented above, DSS-LS rats demonstrated an impaired spatial memory compared to SD-LS rats (Section 2.1.3). The treatment of the DSS rats with MBG or HS did not change their performance in the MWM test. A similar pattern was observed in the SD groups, i.e., none of the stressors affected results of the SD rats (SD-MBG or SD-HS) in the MWM test (Figure 7). All DSS rats, i.e., the DSS-LS, DSS-MBG, and DSS-HS groups, traveled significantly longer distances to find the invisible platform than the SD rats with the corresponding treatment. SD rats showed stronger transfer between visible (trial 9) and hidden (trial 10) platform trials, suggesting better learning and memory for the escape location (Figure 7a). DSS rats in the three experimental groups were less efficient in finding the hidden platform, i.e., they exhibited a decrease in path efficiency in trial 10 (invisible) vs. trial 9 (visible), unlike the corresponding SD groups (Figure 7b) (for details, see the Methods, Section 5.5).

The density of neurons in the hippocampus was estimated in all the studied groups. DSS-LS rats had significantly lower neuronal density in the hippocampus vs. SD-LS rats, as presented in Figure 3, Section 2.1.4. It was further demonstrated that hippocampal neuronal density was not affected by the stressors, neither by MBG nor by HS in both SD and DSS rats in comparison to their LS controls (data are not presented).

## 3. Discussion

In the present study, we demonstrated the following: (**1**) Young DSS rats subjected to an LS intake exhibited higher BP and higher aPWV, i.e., CAS, in comparison to the age- and diet-matched SD-LS rats. The DSS-LS rats displayed upregulated profibrotic signaling in the cardiovascular system and higher collagen abundance in aortic media vs. their SD-LS counterparts. In addition, even on an LS intake, the DSS rats had impaired spatial memory and a lower density of hippocampal neurons vs. SD rats. (**2**) The stimulation of the DSS rats and SD rats with an HS intake or with MBG increased aPWV, activated profibrotic signaling, and increased aortic collagen levels in both strains but was not associated with spatial memory changes either in DSS rats or SD rats. (**3**) In DSS rats, MBG administration was associated with the downregulation of genes related to AD, i.e., APP, PSEN-1, and APOE; this phenomenon was not observed in SD rats.

### 3.1. Dahl Salt-Sensitive Rats as a Model of Vascular Dementia

We compared young DSS rats with age-matched SD rats. More than half a century ago, SD rats were used by Lewis Dahl [5,6] to develop a DSS strain with a hypersensitivity to HS intake. The DSS strain can be considered a mutant strain of SD [5,39]. Both strains carry genome diversity, which was achieved by breeding for the trait of salt sensitivity [6]. It was previously demonstrated that DSS rats exhibited a gradual BP increase with age on an LS intake [40] or normal NaCl intake vs. normotensive rats [41]. Young DSS rats (2.5–3 months old) exhibited an SBP 36 mmHg higher than age-matched DSR rats on an LS diet (0.13%) [42]. That phenomenon was similar to the difference in SBP in DSS rats on an LS diet (0.1%) vs. age- and diet-matched SD rats in the present study. The difference in SBP between four-month-old DSS and SD rats in our study was 26 mmHg, which is in accordance with the previous observations cited above.

We demonstrated in the present study for the first time that DSS rats, being a mutant strain of SD rats, are characterized by higher CAS and lower spatial memory vs. SD rats even on an LS intake at four months of age. It was reported previously that DSS rats subjected to HS intake exhibited higher aPWV vs. DSS rats on an LS diet [3], which is anticipated in the DSS model because of the activation of profibrotic signaling by HS stimulation. Our finding that DSS rats on an LS diet had higher aPWV and a higher SBP than SD rats subjected to LS intake indicates that prohypertensive and profibrotic processes are controlled by factors that are already activated in DSS rats at the age of four months, and which can be further accelerated by the stressor factors. Notably, young DSS rats on an LS diet had higher aPWV in comparison to DSR rats kept on the same diet [43]. Among the known prohypertensive and profibrotic factors are the renin–angiotensin system (RAS), which is corrupted in the DSS rat model [44], and MBG, which participates in the development of hypertension in DSS rats [4]. MGB activates profibrotic signaling via binding to its receptor, NKA [3,15,33]. Previously, we demonstrated that cardiovascular NKA in DSS rats is more sensitive to MBG [2], which allows for a lower concentration of circulating NKA inhibitor to exhibit its profibrotic activity and to activate the downstream pathways. In the present study, we observed that plasma MBG levels were similar in SD-LS and DSS-LS rats (Table 2); nevertheless, DSS-LS rats exhibited higher BP, CAS, and cardiovascular tissue remodeling in comparison to their SD-LS counterparts. We conclude that MBG and MBG-dependent mechanisms may be responsible for the differences between DSS and SD rats at a young age subjected to LS intake, although the other profibrotic and prohypertensive factors, i.e., RAS and particularly angiotensin II (ANGII) [3,45], can also underlie the phenotype difference between these two strains. ANGII stimulates the TGFβ profibrotic signaling cascade in the cardiovascular system in DSS rats [46]. Although we have previously found that ANGII stimulates MBG production via AT1 receptors [45], the complex relations between these two profibrotic systems, i.e., RAS and the NKA–MBG network, and their effect on cardiovascular fibrosis, merit further investigation.

**Figure 8:**
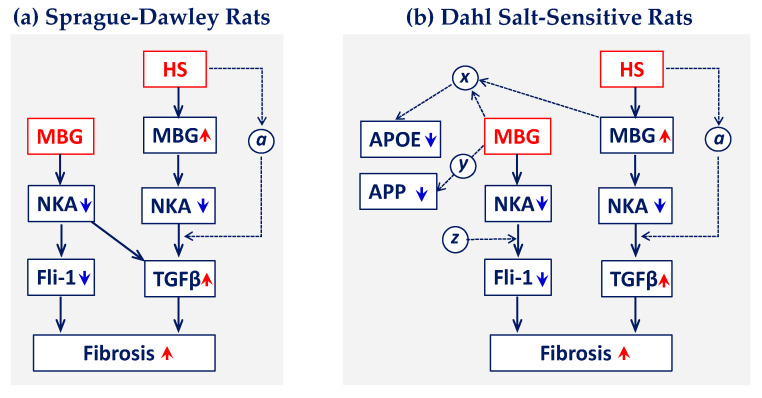
Schematic presentation of the MBG and HS effects on profibrotic signaling in Sprague–Dawley (SD) rats (**a**) and Dahl salt-sensitive (DSS) rats (**b**). HS diet had a similar effect on cardiovascular fibrosis in SD and DSS rats with an activation of TGFβ signaling, whereas MBG administration had a different effect on the genes participating in profibrotic pathways. In SD rats, MBG activated both Fli-1 and TGFβ dependent pathways, whereas in DSS rats, MBG activated only Fli1-dependent profibrotic signaling. In addition, MBG downregulated expression of APOE and APP genes in DSS rats. HS, high salt; MBG, marinobufagenin; NKA, Na/K-ATPase; Fli-1, Freund leukemia integration 1 transcription factor; TGFβ, transforming growth factor beta; APOE, apoliporpotein E; APP, amyloid precursor protein; ***a***, the factor which participates in the activation of TGFβ pathway by an HS diet; ***x***, the factor, which participates in APOE pathway downstream of MBG; ***y***, the factor which participates in APP pathway downstream of MBG; ***z***, the factor which participates in Fli-1 pathway activation and possibly blocks TGFβ pathway. The hypothetical factors ***a***, ***x***, ***y*** and ***z*** require additional investigation; factor ***a*** may be present in both SD and DSS rats, and it is regulated by HS intake; factors ***x***, ***y*** and ***z*** may be activated by MBG in DS rats.

It is known that multiple genes and pathways are involved in the development of hypertension and cardiovascular remodeling in DSS rats [20,47,48,49,50]. Nevertheless, the difference in the genetic profiles between DSS rats and their ancestor SD strain, especially in the absence of HS intake, was not studied in detail. In the present study, we demonstrated that DSS-LS rats in comparison to SD-LS rats have upregulated cardiovascular genes involved in profibrotic signaling, i.e., several collagen types, CTGF, fibronectin, and downregulated Fli1 (Table 3), which is an upstream negative regulator of the collagen gene promoter [15,33,34,50] (Figure 8). The degradation of Fli1 results in increased collagen synthesis and fibrosis. We have previously demonstrated that the Fli1 pathway is a predominant form of profibrotic signaling in the animal model of cardiomyopathy and that Fli1-dependent signaling can be activated by MBG [33,34]. Thus, MBG can directly stimulate rat cardiac fibroblasts to produce collagen, leading to the development of cardiac fibrosis observed with experimental renal failure [15,33]. The incubation of the rat vascular explants with MBG led to the degradation of Fli1 followed by an increase in collagen 1 protein in the aortic media [51]. The incubation of LV myocytes from DSS rats with physiological, i.e., nanomolar, concentrations of MBG was accompanied by a decrease in Fli-1 and increase in collagen 1 [3]. In the present study, LV *Fli-1* mRNA was significantly downregulated in DSS-LS vs. SD-LS rats, which indicates that DSS rats are predisposed to the activation of the Fli1-dependent profibrotic mechanism even in the absence of HS intake. The upregulation of CTGF, FN-1 and several collagens, including Col1a2, was observed in DSS-LS rats in comparison with SD-LS rats (Table 3), which is a characteristic of tissue fibrosis development. The presence of fibrotic changes in the cardiovascular system was supported by the higher aortic weight and higher aortic wall collagen abundance in DSS-LS vs. SD-LS rats.

In the present study, cardiac *APOE* and *APP* mRNA expression was higher in DSS-LS vs. SD-LS rats (Table 3). APOE is implicated in CVD and in AD and is involved in amyloid-β plaque formation [35]. APOE deficiency in the APOE-knockout mouse model is associated with impaired memory and learning ability [52,53]. Previously, it was demonstrated that the association of APOE and AD is dependent on the APOE isoform [54]. Increased *APOE* gene expression may be a biomarker of neurodegeneration. It was demonstrated that saturated lipids contained in APOE lipoparticles mediate astrocyte-induced toxicity which kills neurons in neurodegenerative diseases [55]. The *APOE* polymorphism is a risk factor, not only for AD, but also for dilated cardiomyopathy [56] and hypertension [57]. The observed upregulation of *APOE* mRNA in young DSS rats in comparison to SD rats may be explained by the activation of adaptation to the developing cardiovascular disorder in the DSS rats. 

Another interesting finding in the present study was the upregulated cardiovascular *APP* gene in young DSS rats vs. age-matched SD rats (Table 3). Although APP and amyloid-β protein and their gene expression have been extensively studied in the last few decades, the data on the function of APP in “non-Alzheimer’s” conditions, i.e., without the pronounced brain amyloid-β plaque load, are scant. In a mouse AD model, the cognitive decline was observed prior the formation of the brain amyloid-β plaques [58]. Moreover, a recent clinical study showed cardiac abnormalities in AD patients, suggesting amyloid-β deposition in the heart [59]. Participation of amyloid-β in the development of coronary atherosclerosis and its possible implication in the pathophysiology of cardiovascular diseases are outlined in the review [60]. Interestingly, the function of the full length APP protein outside the central nervous system remains unclear [61]. Thus, the activation of LV *APP* mRNA in the DSS rats in comparison to their normotensive counterparts SD is a novel finding, which required additional investigation of the underlying signaling pathways and the possible mechanistic links between APP and the salt-sensitive genotype in DSS rats.

Young DSS-LS rats exhibited compromised hippocampal spatial memory in comparison to age-matched SD-LS rats, as found using the MWM test in the present study (Figure 3). It has been previously demonstrated that young DSS rats exhibit cognitive dysfunction with LS intake [29,30]. The MWM behavioral test detected significant differences in spatial learning and memory between DSS rats and DSR rats [29,31], which supported the observation in the present study but in comparison of DSS rats to SD rats. In contrast, salt restriction in DSS rats vs. their DSR counterparts did not affect spatial memory but impaired social recognition memory [32]. A passive avoidance test revealed cognitive decline in young DSS-HS rats vs. DSS-LS rats, which was ameliorated by antihypertensive treatment [30]. Thus, DSS rats demonstrated impaired brain function in different cognitive domains vs. DSR rats, which are the genetic salt-resistant antipode of DSS rats [5,6]. The brain function of DSS rats has never been compared to their ancestor SD, as assessed in the present study. The compromised hippocampal spatial memory in DSS-LS rats can be explained by the early development of cardiovascular remodeling and stiffness, which is characterized by higher SBP, aPWV, heart weight, aorta weight, and collagen abundance in the aortic wall in comparison to SD-LS rats. The above cardiovascular changes can contribute to cerebrovascular impairment, which can reduce the blood supply to the brain, which can be followed by alterations to memory and cognitive functions. The underlying mechanisms of these cardiovascular changes may be connected to the impaired ROS in DSS rats [62,63,64]. Finally, we cannot exclude the possible input of the elevated *APP* mRNA on the reduced hippocampal spatial memory in DSS in comparison to SD-LS rats. These mechanisms will be further investigated in the DSS model of vascular dementia.

### 3.2. Effect of the Profibrotic Stressors on Cardiovascular and Behavioral Functions and Gene Expression in Young DSS Rats and SD Rats

Both DSS and SD rats display similar effects from the stressors, MBG and HS diet, on cardiovascular parameters, i.e., SBP, aPWV, aortic wall collagen abundance (Figure 4 and Figure 5), and relative weights of aortae (Table 2). These changes are associated with an MBG increase in the plasma and urine of both DSS and SD rats. The profibrotic effect of MBG has been described in several studies [3]. The MBG infusion in normotensive rats caused cardiovascular fibrosis remodeling, changes in the profibrotic markers associated with this remodeling, and BP changes [15]. The treatment of DSS rats subjected to HS intake with a highly specific anti-MBG monoclonal antibody reduced the circulating MBG level and lowered SBP and aPWV [3], very likely due to the decrease in MBG. Finally, in our previous clinical studies, LV mass was positively and independently associated with MBG excretion in young healthy adults with excessively high MBG levels [65]. Heightened endogenous MBG levels may contribute to high artery stiffness, i.e., higher aPWV, in women via pressure-independent mechanisms, increasing their risk for future cardiovascular disease [66]. In this African-PREDICT study, the regression analysis revealed a positive association between MBG and aPWV [66]. The pressure-independent nature of aPWV and close association of aPWV with MBG was demonstrated in our previous studies [67,68,69], which support our present observation that a similar increase in aPWV and aortic collagen abundance in SD and DSS rats after the treatment with the both stressors was accompanied by moderate BP changes in SD rats and a pronounced hypertensive effect in DSS rats.

Pretreatment of cultured LV myocytes from DSS rats [3], aortic explants [10] and vascular smooth muscle cells from SD [68] rats with a nanomolar concentration of MBG stimulated TGFβ- and/or Fli1-dependent profibrotic signaling, accompanied by a concomitant increase in the levels of collagen. These previous findings provide mechanistic support to the present observations related to the profibrotic activity of MBG. The novelty of the present study is in the comparison of the effects of MBG and HS in DSS rats and in SD rats, a wild type of DSS strain. The profibrotic, prohypertensive, and cardiovascular remodeling effects of the stressors were similar in both strains, although the magnitude of the changes was higher in DSS in comparison to SD rats (Figure 8). An HS diet induced hypertension and cardiac remodeling, resulting in congestive heart failure [3,16], cardiovascular and renal fibrosis [3,17], and stroke in young DSS rats [18,19,20]. Moreover, the HS diet affected CAS in SD rats as well. HS intake in young normotensive SD rats induced vascular fibrosis via pressure-independent/MBG-dependent mechanisms. This remodeling was reduced by immunoneutralization of MBG [70]. Interestingly, the administration of MBG and HS was associated with the stimulation of different profibrotic gene profiles in SD and DSS rats. MBG administration was associated with the upregulation of TGFβ, the downregulation of the *Fli-1* gene and the upregulation of the *col-1a1* gene, which indicates activation of both TGFβ- and Fli-1 dependent profibrotic pathways by MBG in normotensive SD rats (Table 4). HS administration was associated with predominant activation of TGFβ signaling, which was accompanied by activation of some genes, controlled collagen synthesis and Ctgf (Table 4, Figure 6 and Figure 8a). In contrast, MBG administration in DSS rats led to the activation of Fli-1 dependent profibrotic signaling with an activation of several collagen-controlling genes (Table 5). HS diet in DSS rats predominantly activated TGFβ signaling which led to the activation of collagens and Ctgf (Table 5, Figure 6 and Figure 8b).

In the present study, we demonstrated for the first time that young DSS rats have upregulated APP and APOE genes in comparison to age-matched SD rats (Table 3) and that MBG infusion to DSS rats significantly reduced the expression of both genes. An HS diet affected the expression of both APOE and APP genes antithetically in DSS rats (Table 5). No effect of MBG or an HS diet on the above genes was observed in SD rats (Table 4 and Table 5; Figure 6 and Figure 8). It is known that cardenolide NKA inhibitors can prevent amyloid β1–42 aggregation by blocking redox sites and β sheet formation, as was demonstrated in silico [71]. Plant extract of cardenolides provided neuroprotection in organotypic brain slice models for neurodegeneration driven by APP and to ischemic injury modeled by oxygen-glucose deprivation [72]. Nevertheless, there are no publications related to the effect of cardiotonic steroids on AD-related gene expression. Our novel finding of the downregulation of the *APP* mRNA expression in vivo in DSS rats after MBG infusion requires additional investigation of the underlying signaling pathways and the possible mechanistic links between MBG and APP.

The important observation of the present study is that spatial memory was not affected by HS diet or MBG treatment, either in SD or in DSS rats, despite the significant cardiovascular changes, including an increase in CAS and BP. The behavioral and cognitive effects of the chronic MBG infusion have never been studied in rodent models in vivo. The acute administration of MBG attenuated alcohol-seeking behavior in mice [73]. The bufadienolide steroid bufalin, administered acutely to young SD rats, did not cause any changes in the open field test results, although it affected the inflammatory pathways and pain response [74]. Previously, it was demonstrated that cardiotonic steroids, including cardiotonic ouabain, participate in the pathogenesis of depressive disorders [75]. The involvement of the Na/K-ATPase-endogenous cardiac steroids system in bipolar disease has been proven in another study by this group [76]. It was found that the brain level of an endogenous MBG was lower in patients with bipolar disease in comparison to control subjects [76]. Interestingly, in the present study we also observed a lower level of the endogenous MBG in brain tissue in DSS rats vs. SD rats on an LS diet (Table 1). Whether the lower brain MBG level is associated with a moderate lower spatial memory in DSS vs. SD rats will be investigated in future studies. Notably, MBG infusion and HS diet led to an increase in brain MBG levels in both strains (Table 1), without changing the spatial memory (Figure 7). Behavioral tests that probe different cognitive domains merit future investigation of the DSS model of vascular dementia.

In the present study, the DSS-LS group demonstrated spatial memory impairment in the presence of higher SBP and aPWV, activated LV pro-fibrotic signaling, and aortic fibrosis vs. normotensive SD-LS at four months of age, which indicates that DSS-LS rats may develop their cognitive and cardiovascular disfunction before four months of age. HS intake and MBG further increase pro-fibrotic changes in the cardiovascular system. Interestingly, collagen accumulation in the arterial wall of the cerebral arteries was not affected by MBG in both SD and DSS rats, and by HS in SD vs. low salt control groups. Significant collagen abundance only increased in the DSS-HS group vs. DSS-LS (Supplementary Materials, Figure S2). HS intake in DSS rats activates matrix metalloproteinases in the brain that lead to leakage in the blood-brain barrier [30]. An increase in brain vascular fibrosis in DSS-HS may be linked to the counterbalancing of high BP and may partially prevent the rupture of the vessels in the initial stages of hypertension, and thus help to deliver blood to the brain without microbleeds and blood-brain barrier disruption. Although both stressors, HS and MBG, had a pro-fibrotic effect on the aorta in both strains, we did not observe an increase in small cerebral artery fibrosis in both the MBG-treated group or in the DSS-MBG, perhaps due to a different amplitude of the hypertensive responses between treatments or other regulatory factors. On the other hand, an HS diet and MBG infusion increased BP and might have improved brain perfusion that counterbalanced the effect of vascular fibrosis on spatial memory, at least in the initial stages of cardiovascular disorder in young rats. In addition, DSS-LS rats have significantly higher *APP*, *APOE*, and *CTGF* mRNA levels than SD-LS. APP triggers a cascade of neurodegenerative events, including synaptic dysfunction, synaptic loss, formation of intra-neuronal fibrillary tangles, and subsequent neuronal death [77]. APOE is another marker of neurodegeneration [55]. Interestingly, in our study, *APOE* mRNA was upregulated 1.8-fold in DSS-LS group vs. the SD-LS group. However, treatment with HS intake and MBG in DSS rats downregulated *APOE* mRNA. This might partially explain why there was no further decline in spatial memory in DSS rats treated with HS and MBG. CTGF is a negative regulator of neuronal survival in adult neurogenesis and a pro-apoptotic factor, which can reduce the survival rate of neurons in newborns [78]. *CTGF* mRNA was upregulated 1.5-fold in DSS-LS group vs. the SD-LS group. Treatments were also associated with higher *CTGF* mRNA expression. Finally, TGFb-1, a positive neuronal survival regulator in adult neurogenesis, was not different between the SD-LS and DSS-LS groups [79,80], and it was markedly reduced after HS treatment in SD rats and DSS rats. The balance between neurodegeneration and neurogenesis depends on multiple factors and underlies cognitive function, which requires further investigation in this DSS model.

Several studies have been previously conducted to investigate cognitive function in DSS rats. It was demonstrated that DSS rats exhibit cognitive dysfunction at a young age, even on an LS intake [29,30]. The MWM behavioral test detected significant differences in spatial learning and memory between the DSS and DSR rats [29,31]. The passive avoidance test revealed cognitive decline in young DSS rats after HS vs. LS intake, and this cognitive disfunction was ameliorated by antihypertensive treatment [30]. In contrast, salt restriction in DSS rats vs. their DSR counterparts did not affect spatial memory, but impaired social recognition memory [32]. We demonstrated a difference in MWM spatial memory test between young DSS and SD rats on an LS diet. The absence of a cumulative effect of increased aPWV and BP on spatial memory in DSS rats after HS or MBG administration can be explained by the neuroplasticity, which helped the young animals to sustain the elevated CAS. Aging is one of the factors that may result in subtractive changes to neuronal and cerebrovascular plasticity [81]. Although the association between BP, CAS, and cognitive impairment has been recognized in epidemiological and animal model studies, the mechanistic links between these parameters are not fully understood. Moreover, such mechanistic links between cognitive function and vascular structure/function have not yet been explored in animal models. The DSS rat model may represent a model of vascular dementia [29,30,31,32]. Although hypertension in the DSS rats closely resembles that of dysfunction in humans, in which cardiovascular diseases often lead to cognitive decline due to vascular and microvascular diseases, the studies in the DSS rat model as a model of vascular dementia are scarce and merit future development.

## 4. Conclusions

Thus, the prohypertensive and profibrotic effect of HS can be at least partially attributed to the MBG increase in the present study. The prohypertensive and profibrotic functions of MBG were pronounced in both DSS and SD rats, although quantitative PCR revealed different profiles of profibrotic genes in DSS and SD rats activated by MBG or HS. Even on a low salt intake, an overall higher BP, CAS, and cardiovascular remodeling in young DSS rats may underlie their impaired cognitive functions in comparison to the young SD rats. MBG and HS had near similar effects on the cardiovascular system and its function in DSS and SD rats, although the rate of change was lower in SD rats than in DSS rats. In the DSS rats with an HS intake or with MBG administration, the absence of a cumulative effect of higher aPWV and BP on spatial memory can be explained by brain plasticity in young rats, which probably helped the animals to sustain the elevated CAS and to counterbalance the profibrotic effect of MBG.

## 5. Materials and Methods

### 5.1. Animal Experimental Design

The experimental protocol was approved by the Animal Care and Use Committees of the National Institute on Aging (NIA, NIH). Animal care followed the NIH Guide for the Care and Use of Laboratory Animals. Eight-week-old male DSS and SD rats (Charles River, Frederick, MD, USA) were maintained in a temperature-controlled room regulated (26 °C) on a 12 h light/dark cycle with free access to water. SD and DSS rats were randomly selected to receive a rodent diet containing HS (8% NaCl, Harlan Teklad, Madison, WI, USA; *n* = 18/18) or LS (0.1% NaCl, Harlan Teklad, Madison, WI, USA; *n* = 36/29) for 8 weeks. Subgroups of animals on an LS diet were administered MBG (50 μg/kg/day; *n* = 18/15) via osmotic Alzet (DURECT, Cupertino, CA, USA) minipumps for the last 4 weeks of the study (Figure 1, Results section) as described [15]. The following 6 groups of rats were studied: SD-LS, SD-MBG, SD-HS, DSS-LS, DSS-MBG, and DSS-HS. Body weight, SBP, ECHO measurements, and 24 h urine collection were performed at week 8 of the experiment.

For urine collection, animals were placed in metabolic cages (Lab Products, Inc., Seaford, DE, USA) for 24 h, and urine aliquots were kept frozen for subsequent measurement of MBG and creatinine. All animals were tested with the water maze test at the end of the experiment, after which the rats were sacrificed by exsanguination from the abdominal aorta under deep anesthesia by ketamine (100 mg/kg) and xylazine (5 mg/kg). Blood, plasma, thoracic aortae, LV, kidneys, and brains were collected. Heart, LV, kidney, and aorta weights were expressed in milligrams per 100 g of body weight. Aortic weights were normalized with the total length of the thoracic aortae. Aortic pieces and the right brain hemispheres were fixed in 4% formalin buffer solution for histochemical analysis. The left ventricles of the hearts and brains were frozen and kept at −80 °C for further qPCR and MBG measurement.

### 5.2. Marinobufagenin Administration

Osmotic minipumps (Alzet, 2ML4, DURECT, Cupertino, CA, USA) designed to deliver their contents at 2.5 μL/h for 4 weeks were implanted under isoflurane anesthesia (2.5% in oxygen) slightly posterior to the scapulae under the loose skin on the back of the rat at week 4 of the experiment (Figure 1). Minipump units were filled with 2 mL of the vehicle (30% ethyl alcohol and 20% DMSO in 0.9% NaCl) or MBG dissolved in the vehicle solution to deliver 50 µg/kg/day [15]. This dose is closely related to what was found endogenously in rats in a pathological context.

### 5.3. Systolic Blood Pressure and Heart Rate Measurement

Systolic blood pressure (SBP) and heart rate (HR) were measured noninvasively by tail-cuff plethysmography using the MRBP system (IITC Life Science, Woodland Hills, CA, USA) in conscious restrained rats at week 8 of the experiment. The rats were habituated to the experimental environment by training to be restrained in a plastic tube with a tail cuff on and placed in the chamber at 32 °C for up to 30 min for two consecutive days. Five consecutive measurements were performed after 5 min of the acclimation with a 1-min interval. Then, the animal was rested for 5 min, followed by five consecutive BP measurements. The measurement was repeated if necessary to obtain stable readings. For each animal, 10–15 measurements of SBP were performed and the average of at least 5 stable readings was used.

### 5.4. Echocardiography

Evaluation of cardiac function and morphology was performed by transthoracic echocardiography (Sonos 5500, Hewlett-Packard, Andover, MA, USA) at the end of the experiment in all experimental groups [82]. Anesthesia was maintained by mask inhalation of isoflurane (2.5% of isoflurane inhalant mixed with 1 L/min 100% O_2_). The chest and abdomen were shaved, and the animal was positioned on a heating pad in a supine position. HR was monitored by ECG electrodes. To measure aPWV, PW Doppler images were acquired at the level of the aortic arch and abdominal part of the aorta. The transit time of the flow wave from the upper thoracic aorta to the lower abdominal aorta was calculated as the time difference between the two measurements. The distance between these two measurements was measured and used for the aPWV calculation as mm/transit time. Each measurement of aPWV represents the average of five independent determinations per rat.

For the M-mode recordings, the parasternal short-axis view was used at the level of the papillary muscle. The following M-mode measurements were performed: left ventricular (LV) end-diastolic internal diameter (LVIDd), LV end-systolic internal diameter (LVIDs), interventricular septum thickness at diastole (IVSd) and systole (IVSs), and LV posterior wall thickness at diastole (LVPWd) and systole (LVPWs). From these measurements, the fractional shortening (FS) and ejection fraction (EF) were derived. The percentage of LV fractional shortening was calculated as: fractional shortening = [(LVIDd − LVIDs)/LVIDd] × 100%, ejection fraction (EF = [(LVVold − LVVols)/LVVold] × 100%). Heart mass (LV mass = 1.053 × [(LVIDd + LVPWd + IVSd)3 − LVIDd3] × 0.8) was calculated from the two-dimensional mode image.

### 5.5. Redundant Place-Cue Water Maze

To assess the incidental encoding of spatial location information, a modified version of the water maze task was conducted, the redundant place-cue (RPC) task [83]. To reduce stress, rats were handled for three days prior to testing in a 1.8 m diameter white pool. Water temperature was maintained at 27 ± 0.5 °C and made opaque by the addition of white non-toxic water-base tempera paint. The room contained distinct spatial cues, such as a shelf with plastic bottles, a ladder, a rack with mops, and a large blue curtain.

The RPC task consisted of 17 trials with a 15-s inter-trial interval and a 60-s maximum trial duration. For trials 1–9 in a constant location made and visible by mean of a black cap on the platform rising 3 cm above the water surface. Starting from 10th trial, alternating trials were run without the cap, such that the platform was submerged 1 cm beneath the water surface and not visible on trials 10, 12, 14 and 16.

At the start of each trial, a rat was placed into the tank at one of four entry points: north, south, east or west. The entry point for each trial was semi-randomized across trials, but the order was constant across all animals. No trial had the same entry point as the prior trial, but all rats had the same entry point on any given trial number. If the platform was not located within the allowed time, they were guided by hand to the platform. After each trial, the rat remained on the platform for an additional 20 s. After removal from the tank, rats were manually dried with a paper towel and placed under the red-light lamp.

After completion of the test, all tracks from all trials were analyzed using Anymaze software (Stoelting Co., Wood Dale, IL, USA).

### 5.6. Marinobufagenin Assay

The urine, plasma, and brain MBG concentrations were measured using a competitive fluoro-immunoassay based on a monoclonal murine anti-MBG 4G4 antibody [84]. Plasma was extracted using SepPak C-18 cartridges (Waters Inc., Milford, MA, USA). Brain cortex samples were homogenized in phosphate-buffered saline (PBS) (50 mg/mL), sonicated for 5 s, and centrifuged (5 min, 1000× *g*) to remove tissue debris. The supernatants were used for the protein measurements (Bio-Rad Protein Assay; Bio-Rad/Life Science, Hercules, CA, USA)) and MBG extraction with C18 Sep-Pak cartridges (Waters Inc., Milford, MA, USA), as reported previously [76]. The MBG immunoassay is based on the competition between the immobilized antigen (MBG-glycoside-Thyroglobulin) and MBG within the sample for a limited number of binding sites on the 4G4 monoclonal anti-MBG (against MBG-glycoside-albumin) mouse antibody (1:500). Secondary anti-mouse antibody (1:4000, Perkin Elmer, Waltham, MA, USA) was labeled with non-radioactive europium. The cross-immunoreactivity of 4G4 monoclonal anti-MBG antibody is (in %): MBG, 100; marinobufotoxin, 43; cinobufotalin, 40; telocinobufagin, 14; resibufagenin, 0.5; bufalin, 0.08; cinobufagin, 0.07; digoxin, 0.03; ouabain, 0.005; ouabagenin, 0.001; digoxigenin, 0.004; proscillaridin A, digitoxin, aldosterone, progesterone, prednisone, corticosterone and thyroglobulin, <0.001. MBG (>98% HPLC-pure) was purified from secretions from parotid glands of Bufo marinus toads, as reported previously [3]. Urinary MBG excretion was expressed as pmol per 24 h per 100 g of body weight and brain MBG was expressed as pmol per g protein.

### 5.7. Creatinine Assays

Creatinine and BUN levels were analyzed in the plasma using commercially available i-STAT cartridges Crea and CHEM 8+ (Abbott Point of Care, Princeton, NJ, USA). Urine creatinine was measured by a colorimetric method using a kit (Cayman Chemical, Ann Arbor, MI, USA), according to the provided protocol. Plasma and urine creatinine were used to estimate creatinine clearance (CrCl), which was calculated as CrCl = (uCr × uVol)/(pCr × 1440), where uCr and pCr are urine creatinine and plasma creatinine concentrations (mmol/L), respectively. uVol is the volume of urine in ml produced during 24 h, or 1440 min, and expressed as mL/min.

### 5.8. Histopathological Staining

Formalin-fixed paraffin-embedded aortae were prepared using standard procedures. Thoracic aortic sections (6 μm) were stained with Masson Trichrome (MTC) stain or Verhoeff’s stain (American Master Tech Inc., Lodi, CA, USA) to evaluate the development of fibrosis (in MTC, collagen is stained blue; in Verhoeff’s stain, collagen is stained red, and elastin is stained black). Color images were captured with the ZEISS Axioplan microscope (Carl Zeiss Inc., Thornwood, NY, USA) using a QCAM FAST 1394 digital camera (QImaging, Surrey, BC, Canada). The collagen and elastin amounts were estimated by Metamorph Image Analysis Software (Molecular Devices, LLC, San Jose, CA, USA). In aortic tissues, collagen and elastin were assessed in the media and expressed as percent of the total media area.

Formalin-fixed paraffin-embedded right brain hemispheres were prepared using standard procedures. Parasagittal sections (6 μm) taken 1.5 to 2.5 mm laterally to midsagittal plane were used for the neuronal density quantification. The brain sections were stained with Luxol Fast Blue/Cresyl violet stain (StatLab Medical Products, LLC, McKinney, TX, USA), which allows visualization of neuroanatomic structures based on their degree of myelination (neurons stained dark blue and purple). Both pyramidal cells and neurons were quantified in selected CA1 and CA3 regions and the area of CA1 and CA3 were also estimated in mm^2^ (for details, see also the legend to Figure 4). Color images were captured with the ZEISS Axioplan fluorescence microscope using a QCAM FAST 1394 digital camera and evaluated using the Metamorph Image Analysis Software (Molecular Devices, LLC, San Jose, CA, USA). The density of the neurons was expressed as number of the neurons per mm^2^ of the selected area. The brain sections (6 µm thick) were also stained with PicroSirius Red/Fast Green (Chondrex, Inc., Redmond, WA, USA) to visualize collagen in cerebral arterial media. The slides were then dehydrated three times in 100% ethanol, cleared in xylene, and mounted with Permount (Fisher Scientific, Asheville, NC, USA). For quantitative image analysis, the MetaMorph Image Analysis Software (Molecular Devices, LLC, San Jose, CA, USA) was used. In the cerebral arteries, collagen was assessed in the media and expressed as percent of the total media area.

### 5.9. Total RNA Purification and Real-Time Quantitative PCR

The real-time qPCR analysis of *COL1a1*, *COL1a2*, *COL3a1*, *COL4a1*, *COL5a1*, *TGFb1*, *CTGF1*, *FN1*, *FLI1*, *APOE*, *APP*, *PSEN1*, and *PSEN2* mRNA levels in LV was performed by the amplification of the resulting cDNAs and was normalized to the expression of the housekeeping gene (glyceraldehyde-3-phosphate dehydrogenase (*GAPDH*)) mRNA as an internal standard. Total RNA was purified from the LV (30–35 μg/sample) using the Qiagen RNeasy mini-kit (Qiagen Inc., Germantown, MD, USA), and the RNA samples were reverse-transcribed to cDNA using the QuantiTech Reverse transcription kit (Qiagen Inc., Germantown, MD, USA). Information for the gene primers (Qiagen Inc., Germantown, MD, USA) used for qPCR is presented in the Supplementary Table S1. QPCR was performed with a QuantiFast SYBR Green PCR kit (Qiagen Inc., Germantown, MD, USA), in accordance with the manufacturer’s protocol, with an Applied Biosystems Quant Studio 3 Real-Time PCR Instrument (Applied Biosystems, Grand Island, NY, USA).

Gene expression was analyzed in each sample by the following protocol: activation at 95 °C (8 min) followed by 40 cycles consisting of a first phase of denaturation at 95 °C (10 s), and a second phase of annealing/extending at 60 °C (30 s). Each reaction was performed in triplicate with the inclusion of non-template controls in each experiment. A dissociation curve analysis was performed in each experiment to eliminate non-specific amplification, including primer dimers. The *GAPDH C_t_* values were subtracted from the raw sample *C_t_* values to obtain the corrected *C_t_*. Power conversion (power (2^−(corrected *Ct*)^) was used to convert corrected *Ct* to relative RNA quantity.

### 5.10. Statistical Analysis

Results are presented as mean ± standard error of the mean (SEM). Shapiro–Wilk normality tests (GraphPad Prism 8 software; GraphPad Inc., La Jolla, CA, USA) were conducted for each sample and each variable. All samples passed the normality test (*p* > 0.05). Next, the Bartlett test for equal variances detected that the variances did not differ significantly among the groups. Because our data were consistent with normal distributions with constant variance, the parametric ANOVA was applied for data analysis: 2-way ANOVA, followed by Tukey’s post-hoc test for correction for multiple comparison or by Holm–Sidak’s multiple comparison post-hoc test, were used as specified in the table and the figure legends, or the *t*-test where applicable (GraphPad Prism software; GraphPad Inc., La Jolla, CA, USA). A 2-sided *p* value of <0.05 was considered significant.

## Figures and Tables

**Table 1:** Basic parameters in Sprague–Dawley rats and Dahl salt-sensitive rats following a low-salt (LS) diet, marinobufagenin (MBG), or a high-salt (HS) diet administration.

	Sprague–Dawley Rats			Dahl Salt-Sensitive Rats		
	SD-LS (*n* = 18)	SD-MBG (*n* = 18)	SD-HS (*n* = 18)	DSS-LS (*n* = 14)	DSS-MBG (*n* = 15)	DSS-HS (*n* = 18)
**Physiological parameters:**						
Body weight (g)	524 ± 10	531 ± 13	471 ± 8 **	385 ± 6 ##	386 ± 5 ##	323 ± 6 **##
SBP (mmHg)	117 ± 2	132 ± 4 *	137 ± 4 *	143 ± 5 ##	163 ± 5 *##	210 ± 7 **##
HR (beat/min)	388 ± 10	402 ± 9	395 ± 8	410 ± 9 #	404 ± 6	430 ± 10 #
**Biochemical parameters (plasma and brain):**						
Plasma MBG (pmol/L)	334 ± 34	497 ± 57 †	490 ± 64 †	251 ± 39	541 ± 83 *	689 ± 90 **
Plasma Na^+^ (mmol/L)	140.9 ± 0.3	140.7 ± 0.4	141.6 ± 0.5	138.3 ± 1.1	140.5 ± 0.6	141.4 ± 1.0
Plasma K^+^ (mmol/L)	4.57 ± 0.15	4.70 ± 0.24	4.91 ± 0.26	4.88 ± 0.25	5.11 ± 0.28	4.98 ± 0.30
Hematocrit (%)	42.5 ± 0.7	41.7 ± 0.3	45.0 ± 0.7	43.8 ± 0.9	43.8 ± 1.1	37.9 ± 1.5 **##
Plasma Cr (μmol/L)	37.9 ± 1.5	37.8 ± 1.5	33.9 ± 1.8	49.2 ± 3.1 #	49.1 ± 3.4 #	66.0 ± 3.2 **##
BUN (mmol/L)	5.8 ± 0.2	5.5 ± 0.2	5.9 ± 0.3	6.7 ± 0.3	7.4 ± 0.3 #	10.1 ± 0.5 **##
Brain MBG (pmol/g prot)	9.9 ± 0.9	17.3 ± 1.6 **	16.1 ± 2.0 *	3.9 ± 0.5 #	16.9 ± 1.2 **	23.2 ± 1.6 **
**Urinary profile:**						
Urine volume (mL/24 h)	13.4 ± 1.2	13.1 ± 1.4	69.4 ± 3.9 **	10.9 ± 1.1	14.2 ± 1.8	82.8 ± 5.1 **#
Urine Cr (mmol/L)	9.02 ± 0.97	8.31 ± 0.88	1.77 ± 0.09 **	10.00 ± 0.98	6.54 ± 0.80 *	1.32 ± 0.09 **
Cr clearance (ml/min)	2.7 ± 0.3	2.4 ± 0.2	3.0 ± 0.3	2.6 ± 0.3	2.1 ± 0.4	1.3 ± 0.1 *##
Urine MBG (pmol/24 h/kg BW)	48.1 ± 7.1	110.3 ± 12.4 *	146.4 ± 21.1 **	57.0 ± 4.9	91.0 ± 13.4 †	297.6 ± 15.3 **##

Values are expressed as mean ± SEM after MBG administration and after LS and HS diets. Two-way ANOVA followed by Holm-Sidak’s multiple comparison post hoc test: * *p* < 0.05, ** *p* < 0.01 for MBG or HS vs. LS (effect of MBG or HS); # *p* < 0.05, ## *p* < 0.01 for DSS rats vs. SD rats (effect of strain) at each condition. Two-tail unpaired *t*-test: † *p* < 0.05, effect of MBG vs. LS in both strains. BUN, blood urea nitrogen; BW, body weight; Cr, creatinine; HR, heart rate; SBP, systolic blood pressure. **SBP:** Strain effect, *p* < 0.01; MBG effect, *p* < 0.01; HS effect, *p* < 0.01; Strain × MBG, *p* = 0.58; Strain × HS, *p* < 0.01. **HR:** Strain effect, *p* = 0.01; MBG effect *p* = 0.67; HS effect, *p* = 0.16; Strain × MBG, *p* = 0.28; Strain × HS, *p* = 0.50. **Plasma MBG:** Strain effect, *p* = 0.32; MBG effect, *p* < 0.01; HS effect, *p* < 0.01; Strain × MBG, *p* = 0.26; Strain × HS, *p* = 0.03. **Brain MBG:** Strain effect, *p* = 0.88; MBG effect, *p* < 0.01; HS effect, *p* < 0.01; Strain × MBG, *p* < 0.01; Strain × HS, *p* < 0.01. **Urine MBG:** Strain effect, *p* < 0.01; MBG effect, *p* < 0.01; HS effect, *p* < 0.01; Strain × MBG, *p* = 0.18; Strain × HS, *p* < 0.01.

**Table 2:** Echocardiography parameters and tissue weights in Sprague–Dawley rats and Dahl salt-sensitive rats following low-salt (LS) diet, marinobufagenin (MBG) or high-salt (HS) diet administration.

	Sprague–Dawley Rats			Dahl Salt-Sensitive Rats		
	SD-LS (*n* = 18)	SD-MBG (*n* = 18)	SD-HS (*n* = 18)	DSS-LS (*n* = 14)	DSS-MBG (*n* = 15)	DSS-HS (*n* = 18)
**Echocardiography:**						
IVSd (mm)	1.60 ± 0.04	1.42 ± 0.03	1.64 ± 0.04	1.42 ± 0.03	1.53 ± 0.03	1.96 ± 0.07 **##
IVSs (mm)	2.87 ± 0.07	2.95 ± 0.07	3.10 ± 0.05	2.99 ± 0.07	3.08 ± 0.07	3.43 ± 0.10 **#
LVIDd (mm)	7.94 ± 0.08	7.79 ± 0.13	7.83 ± 0.13	7.42 ± 0.12 #	7.43 ± 0.07	6.83 ± 0.07 **##
LVIDs (mm)	4.51 ± 0.13	4.36 ± 0.07	4.43 ± 0.13	3.78 ± 0.21 ##	3.76 ± 0.10 #	3.85 ± 0.10 #
LVPWd (mm)	2.01 ± 0.06	1.94 ± 0.05	2.07 ± 0.06	1.83 ± 0.06	1.90 ± 0.05	2.54 ± 0.09 **##
LVPWs (mm)	3.03 ± 0.07	3.06 ± 0.08	3.26 ± 0.07	3.08 ± 0.09	3.08 ± 0.10	3.58 ± 0.10 **
FS (%)	44.3 ± 1.2	44.0 ± 0.4	43.8 ± 1.1	50.7 ± 2.1 #	48.7 ± 1.3	43.9 ± 1.0 *
EF (%)	79.5 ± 1.4	80.0 ± 1.2	80.2 ± 1.3	85.5 ± 1.7 #	84.5 ± 1.1	80.7 ± 1.1
HR (beat/min)	372 ± 10	364 ± 6	343 ± 11	384 ± 7	379 ± 9	366 ± 6
LV mass, g	1.50 ± 0.02	1.47 ± 0.03	1.55 ± 0.03	1.35 ± 0.03 #	1.35 ± 0.03	1.59 ± 0.05 **
RWT	0.51 ± 0.02	0.50 ± 0.02	0.53 ± 0.02	0.49 ± 0.02	0.51 ± 0.01	0.74 ± 0.03 **##
aPWV (m/s)	2.20 ± 0.09	3.33 ± 0.14 *	4.36 ± 0.25 **	2.51 ± 0.08 †	3.67 ± 0.17 *	5.31 ± 0.28 **#
**Tissue weights:**						
Heart/BW (g/kg)	2.72 ± 0.07	2.67 ± 0.07	3.07 ± 0.08	3.10 ± 0.06	3.18 ± 0.05 ##	4.36 ± 0.18 **##
LV/BW (g/kg)	1.09 ± 0.02	1.04 ± 0.03	1.26 ± 0.03	1.31 ± 0.04 #	1.34 ± 0.04 ##	2.05 ± 0.15 **##
Kidneys/BW (g/kg)	6.7 ± 0.2	6.6 ± 0.1	8.3 ± 0.3 *	7.7 ± 0.3	7.3 ± 0.1	11.4 ± 0.7 **##
Aorta/BW (mg/mm·kg)	2.57 ± 0.06	2.84 ± 0.05 §	3.01 ± 0.06 §	3.64 ± 0.09 ##	3.97 ± 0.11 §##	5.38 ± 0.28 **##

Values are expressed as mean ± SEM after MBG administration and after LS and HS intake. Two-way ANOVA followed by Holm-Sidak’s multiple comparison post hoc test: * *p* < 0.05, ** *p* < 0.01, for MBG and HS vs. LS (effect of MBG or HS); # *p* < 0.05, ## *p* < 0.01, for DSS rats vs. SD rats (effect of strain) at each condition. Two-tail unpaired *t*-test: † *p* < 0.05 for DSS-LS vs. SD-LS; § *p* < 0.05 for effect of MBG or HS vs. LS. BW, body weight; EF, ejection fraction; FS, fractional shortening; HR, heart rate; IVSd and IVSs, interventricular septum at end diastole and end systole; LV, left ventricle; LVIDd and LVIDs, LV internal diameter at end diastole and end systole; LVPWd and LVPWs, LV posterior wall thickness at diastole and systole; LVW, LV weight; aPWV, aortic pulse wave velocity; RWT, relative LV wall thickness. **LVIDs:** Strain effect, *p* < 0.01; MBG effect, *p* < 0.01; HS effect, *p* < 0.01; Strain × MBG, *p* = 0.48; Strain × HS, *p* = 0.02. **LVIDd:** Strain effect, *p* < 0.01; MBG effect, *p* < 0.01; HS effect, *p* < 0.01; Strain × MBG, *p* = 0.64; Strain × HS, *p* = 0.59. **aPWV:** Strain effect, *p* < 0.01; MBG effect, *p* < 0.01; HS effect, *p* < 0.01; Strain × MBG, *p* = 0.92; Strain × HS, *p* = 0.27. **LV/BW:** Strain effect, *p* < 0.01; MBG effect, *p* = 0.75; HS effect, *p* < 0.01; Strain × MBG, *p* = 0.21; Strain × HS, *p* < 0.01. **Kidney/BW:** Strain effect, *p* < 0.01; MBG effect, *p* = 0.77; HS effect, *p* < 0.01; Strain × MBG, *p* = 0.51; Strain × HS, *p* = 0.03. **Aorta/BW:** Strain effect, *p* < 0.01; MBG effect, *p* < 0.01; HS effect, *p* < 0.01; Strain × MBG, *p* = 0.95; Strain × HS, *p* < 0.01.

**Table 3:** Expression of genes associated with cardiovascular diseases, fibrosis, and Alzheimer’s disease in left ventricles from Dahl salt-sensitive rats in comparison to Sprague–Dawley rats on an LS diet (by qPCR test).

Gene Symbol	Description	SD-LS	DSS-LS (Folds to SD-LS)
*APOE*	Apolipoprotein E	1.00 ± 0.09	1.78 ± 0.36 *
*APP*	Amyloid precursor protein	1.00 ± 0.03	2.18 ± 0.42 **
*TGF* *β-1*	Transforming growth factor, beta 1	1.00 ± 0.04	1.04 ± 0.07
*Fli-1*	Freund leukemia integration 1 transcription factor	1.00 ± 0.04	0.80 ± 0.07 *
*Col1a1*	Collagen, type I, alpha 1	1.00 ± 0.04	0.92 ± 0.10
*Col1a2*	Collagen, type I, alpha 2	1.00 ± 0.12	1.90 ± 0.24 **
*Col3a1*	Collagen, type III, alpha 1	1.00 ± 0.13	1.59 ± 0.19 *
*Col4a1*	Collagen, type IV, alpha 1	1.00 ± 0.10	1.93 ± 0.25 **
*Col5a1*	Collagen, type V, alpha 1	1.00 ± 0.07	1.06 ± 0.14
*Col5a2*	Collagen, type V, alpha 2	1.00 ± 0.11	1.41 ± 0.09 *
*Ctgf*	Connective tissue growth factor	1.00 ± 0.10	1.50 ± 0.16 *
*FN-1*	Fibronectin 1	1.00 ± 0.08	1.40 ± 0.07 **
*PSEN-1*	Presenilin 1	1.00 ± 0.04	1.20 ± 0.15
*PSEN-2*	Presenilin 2	1.00 ± 0.06	1.16 ± 0.03

Values are expressed as mean ± SEM. By two-tailed unpaired *t*-test: * *p* < 0.05; ** *p* < 0.01, for DSS vs. SD rats. DSS, Dahl salt-sensitive rats; SD, Sprague–Dawley rats; LS, low salt. Significant changes are presented in bold.

**Table 4:** Expression of fibrosis-associated genes and other genes in the left ventricles of Sprague–Dawley (SD) rats treated with MBG or HS vs. LS control assessed by qPCR.

Gene Symbol	Description	SD-LS	SD-MBG (Folds to SD-LS)	SD-HS (Folds to SD-LS)
*APOE*	Apolipoprotein E	1.00 ± 0.08	1.10 ± 0.18	1.32 ± 0.22
*APP*	Amyloid precursor protein	1.00 ± 0.03	1.02 ± 0.05	1.08 ± 0.06
*TGF* *β* *-1*	Transforming growth factor, beta 1	1.00 ± 0.05	1.33 ± 0.11 *	1.58 ± 0.10 **
*Fli-1*	Freund leukemia integration 1 transcription factor	1.00 ± 0.06	**0.80 ± 0.07 †**	0.98 ± 0.09
*Col1a1*	Collagen, type I, alpha 1	1.00 ± 0.05	1.26 ± 0.16	1.47 ± 0.27
*Col1a2*	Collagen, type I, alpha 2	1.00 ± 0.10	1.47 ± 0.12 **	0.98 ± 0.08
*Col3a1*	Collagen, type III, alpha 1	1.00± 0.07	1.08 ± 0.09	0.97 ± 0.06
*Col4a1*	Collagen, type IV, alpha 1	1.00 ± 0.05	0.95 ± 0.07	0.85 ± 0.09
*Col5a1*	Collagen, type V, alpha 1	1.00 ± 0.05	1.33 ± 0.16	1.51 ± 0.13 *
*Col5a2*	Collagen, type V, alpha 2	1.00 ± 0.07	1.01 ± 0.07	0.81 ± 0.06
*Ctgf*	Connective tissue growth factor	1.00 ± 0.12	1.28 ± 0.20	3.02 ± 0.39 **
*FN-1*	Fibronectin 1	1.00 ± 0.12	1.22 ± 0.13	1.19 ± 0.13
*PSEN-1*	Presenilin 1	1.00 ± 0.03	0.90 ± 0.04	0.79 ± 0.05 **
*PSEN-2*	Presenilin 2	1.00 ± 0.03	1.17 ± 0.09	1.36 ± 0.09 **

Values are expressed as mean ± SEM. One-way ANOVA followed by Tukey’s post hoc test: * *p* < 0.05; ** *p* < 0.01, MBG or HS vs. LS. Two-tailed *t*-test: † *p* < 0.05 vs. LS. LS, low salt; MBG, marinobufagenin; HS, high salt. Significant changes are presented in bold.

**Table 5:** Expression of fibrosis-associated and other genes in left ventricles from **Dahl salt-sensitive** (**DSS**) **rats** treated with MBG or HS vs. LS control by qPCR test.

Gene Symbol	Description	DSS-LS	DSS-MBG (Folds to DSS-LS)	DSS-HS (Folds to DSS-LS)
*APOE*	Apolipoprotein E	1.00 ± 0.08	0.36 ± 0.05 **	0.63 ± 0.09 **
*APP*	Amyloid precursor protein	1.00 ± 0.06	0.53 ± 0.07 **	1.48 ± 0.14 **
*TGF* *β* *-1*	Transforming growth factor, beta 1	1.00 ± 0.03	0.90 ± 0.05	1.59 ± 0.11 **
*Fli-1*	Freund leukemia integration 1 transcription factor	1.00 ± 0.05	0.61 ± 0.03 **	1.05 ± 0.03
*Col1a1*	Collagen, type I, alpha 1	1.00 ± 0.06	0.75 ± 0.04	1.65 ± 0.20 **
*Col1a2*	Collagen, type I, alpha 2	1.00 ± 0.06	2.26 ± 0.21 **	2.80 ± 0.37 **
*Col3a1*	Collagen, type III, alpha 1	1.00 ± 0.06	1.67 ± 0.15 *	2.30 ± 0.24 **
*Col4a1*	Collagen, type IV, alpha 1	1.00 ± 0.04	1.39 ± 0.08 **	1.68 ± 0.10 **
*Col5a1*	Collagen, type V, alpha 1	1.00 ± 0.04	0.90 ± 0.06	1.47 ± 0.09 **
*Col5a2*	Collagen, type V, alpha 2	1.00 ± 0.06	1.05 ± 0.09	1.56 ± 0.10 **
*Ctgf*	Connective tissue growth factor	1.00 ± 0.07	1.33 ± 0.25	4.24 ± 0.65 **
*FN-1*	Fibronectin 1	1.00 ± 0.08	1.26 ± 0.17	2.62 ± 0.14 **
*PSEN-1*	Presenilin 1	1.00 ± 0.06	0.59 ± 0.04 **	0.90 ± 0.07
*PSEN-2*	Presenilin 2	1.00 ± 0.04	1.00 ± 0.10	**1.23 ± 0.10 †**

Values are expressed as mean ± SEM. One-way ANOVA followed by Tukey’s post hoc test: * *p* < 0.05; ** *p* < 0.01, MBG or HS vs. LS. Two-tailed *t*-test: † *p* < 0.05 vs. LS. LS, low salt; MBG, marinobufagenin; HS, high salt. Significant changes are presented in bold.

## Data Availability

The data presented in this study are available from the corresponding author upon reasonable request.

## Supplementary Materials

Supplementary Materials can be found at https://www.mdpi.com/article/10.3390/ijms23094563/s1.

## Abbreviations

aPWV	Aortic pulse wave velocity
CAS	Central arterial stiffness
CVD	Cardiovascular disease
DELFIA	Dissociation-enhanced lanthanide fluorescence immunoassay
DSS	Dahl salt-sensitive
DSR	Dahl salt-resistant
ECHO	Echocardiography
EF	Ejection fraction
Fli-1	Freund leukemia integration 1 transcription factor
FS	Fractional shortening
HS	High salt
LS	Low salt
LV	Left ventricle
LVIDd	Left ventricle internal diameters at end diastole
LVIDs	Left ventricle internal diameters at end systole
MBG	Marinobufagenin
MWM	Morris water maze
NKA	Na/K-ATPase
PCR	Polymerase chain reaction
ROS	Reactive Oxygen Spices
SBP	Systolic blood pressure
SD	Sprague–Dawley
TGFb	Transforming growth factor, beta 1
