# Supplementary material for: Effect of Cardiotonic Steroid Marinobufagenin on Vascular Remodeling and Cognitive Impairment in Young Dahl-S Rats
Source: Int J Mol Sci. 2022 Apr 20;23(9):4563. doi: 10.3390/ijms23094563 (PMC9101263; doi:10.3390/ijms23094563)
Supplement: Supplementary file 1 [file ijms-23-04563-s001.zip › ijms-1605037-SM.pdf]

International Journal of *Molecular Sciences*

MDPI

Supplementary Materials

Table S1. Primers used for quantitative real-time polymerase chain reaction analysis.

Gene symbol and name	QuantiTect Primer Assay name (Qiagen)
APOE rat (apolipoprotein E)	Rn_Apoe_1_SG
APP rat (amyloid precursor protein)	Rn_App_1_SG
COL1a1 rat (collagen 1 alpha 1)	Rn_Col1a1_2_SG
COL1a2 rat (collagen 1 alpha 2)	Rn_Col1a2_2_SG
COL3a1 rat (collagen 3 alpha 1)	Rn_Col3a1_1_SG
COL4a1 rat (collagen 4 alpha 1)	Rn_Col4a1_1_SG
COL5a1 rat (collagen 5 alpha 1)	Rn_Col5a1_1_SG
CTGF rat (connective tissue growth factor 1)	Rn_Ctgf_1_SG
FLI1 rat (Friend leukemia integration 1 transcription factor)	Rn_Fli1_1_SG
FN1 rat (fibronectin 1)	Rn_Fn1_1_SG
GAPDH rat (glyceraldehydes-3-phosphate dehydrogenase)	Rn_Gapdh_1_SG
PSEN1 rat (presenilin 1)	Rn_PSEN1_1_SG
PSEN2 rat (presenilin 2)	Rn_RGD:621060_1_SG
TGFb1 rat (transforming growth factor beta 1)	Rn_Tgfb1_1_SG

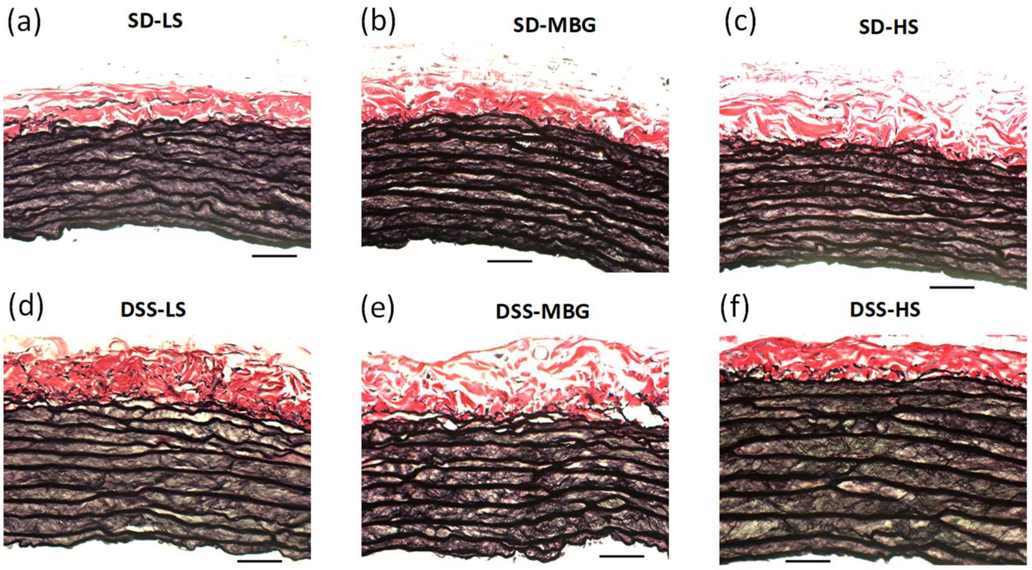

Figure S1. Collagen and elastin in rat aortae. (a-f) The representative photomicrographs of aortae stained by Verhoeff's stain (collagen is stained red, elastin is stained black) from each experimental group: (**a**) SD-LS; **(b)** SD-MBG; **(c)** SD-HS; **(d)** DSS-LS; **(e)** DSS-MBG; **(f)** DSS-HS. The difference in elastin abundance was non-significant between all groups. SD, Sprague-Dawley rats; DSS, Dahl salt sensitive rats; LS, low salt diet; MBG, marinobufagenin; HS, high salt diet. Scale bar is 50 μm.

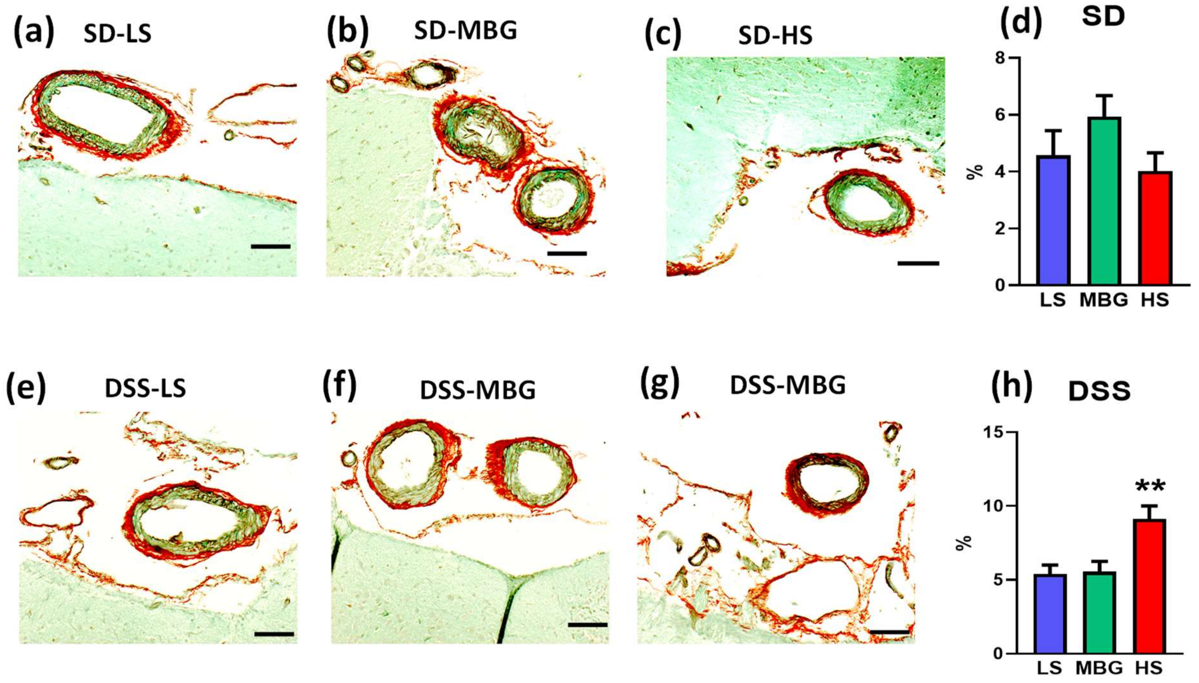

Figure S2. Collagen in arterial media in cerebral arteries. Representative photomicrographs of cerebral arteries from SD **(a-c)** and DSS **(e-g)** rats stained with Picro-Sirius Red Fast Green; collagen abundance in the arterial media in SD **(d)** and DSS **(h).** Collagen in the aortic wall is stained red; muscle and intracellular fibers are stained green (*n*= 6 rats/group; 2–7 images of arteries per rat). SD-LS **(a),** SD-MBG **(b),** SD-HS **(c),** DSS-LS **(e),** DSS-MBG **(f),** DSS-HS **(g),** SD **(d),** DSS **(h).** By 2-way ANOVA: $^{**}p < 0.01$ vs. LS. SD, Sprague-Dawley rats; DSS, Dahl salt-sensitive rats; LS, low-salt diet; MBG, marinobufagenin; HS, high-salt diet. Scale bar is 100 μm.
